# The complementary roles of STAT3 and STAT1 in cancer biology: insights into tumor pathogenesis and therapeutic strategies

**DOI:** 10.3389/fimmu.2023.1265818

**Published:** 2023-11-08

**Authors:** Weiyuan Wang, Melanie Cristina Lopez McDonald, Christine Kim, Mirielle Ma, Zetao (Tommy) Pan, Charlotte Kaufmann, David A. Frank

**Affiliations:** Department of Hematology and Medical Oncology, Winship Cancer Institute, Emory University School of Medicine, Atlanta, GA, United States

**Keywords:** cancer, oncology, transcription factors, signal transduction, tumor immunology, protein phosphorylation

## Abstract

STATs are a family of transcription factors that regulate many critical cellular processes such as proliferation, apoptosis, and differentiation. Dysregulation of STATs is frequently observed in tumors and can directly drive cancer pathogenesis. STAT1 and STAT3 are generally viewed as mediating opposite roles in cancer development, with STAT1 suppressing tumorigenesis and STAT3 promoting oncogenesis. In this review, we investigate the specific roles of STAT1 and STAT3 in normal physiology and cancer biology, explore their interactions with each other, and offer insights into therapeutic strategies through modulating their transcriptional activity.

## 1 Introduction

Cellular phenotype is ultimately driven by the pattern of gene expression within a given cell. Genes regulating critical cellular functions such as survival, proliferation, and self-renewal often code for transcripts with short half-lives, so that the initiation of transcription is a major control point in their expression. Thus, the activity of transcription factors, which ultimately coordinate gene expression, is highly regulated. Conversely, inappropriate or constitutive activation of transcription factors, due to over-expression, increased activity from post-translational modifications, or direct mutation, is a common event driving malignancy. A number of these so-called oncogenic transcription factors have been described, and have been the focus of basic, translational, and therapeutic studies. However, transcription factors often interact with each other either through direct protein-protein interactions, in higher order structures on chromatin, or through functional networks (1). Therefore, to understand the biology of oncogenic transcription factors in cancer pathogenesis or as targets for therapy, it is important to consider their activity in the context of other transcriptional regulators that may be co-expressed.

## 2 The STAT family of transcription factors

Signal transducer and activator of transcription (STAT) proteins are transcription factors that mediate many critical aspects of cellular function, including proliferation, apoptosis, and differentiation. There are seven members in the mammalian STAT family: STAT1, STAT2, STAT3, STAT4, STAT5 (STAT5A and STAT5B), and STAT6. These proteins share common structural motifs, including an N-terminal domain followed by a coiled-coil domain, DNA-binding domain, linker, Src homology 2 (SH2) domain, and a C-terminal transactivation domain.

The canonical transcriptional activity of STATs is triggered by the phosphorylation of a single tyrosine located towards the carboxy terminus of all STAT family members. While STATs are largely found as inactive dimers in the cytoplasm under basal conditions, this tyrosine phosphorylation leads to a conformational change in the STAT dimer mediated by reciprocal phosphotyrosine-SH2 interactions. This form of the dimer reveals a nuclear localization signal, triggering translocation to the nucleus. Most STATs then bind nine base pair DNA sequences of the general form TTCN_3_GAA, from which they can trigger transcriptional activation (or, in some case, repression) of target genes.

**Figure 1:**
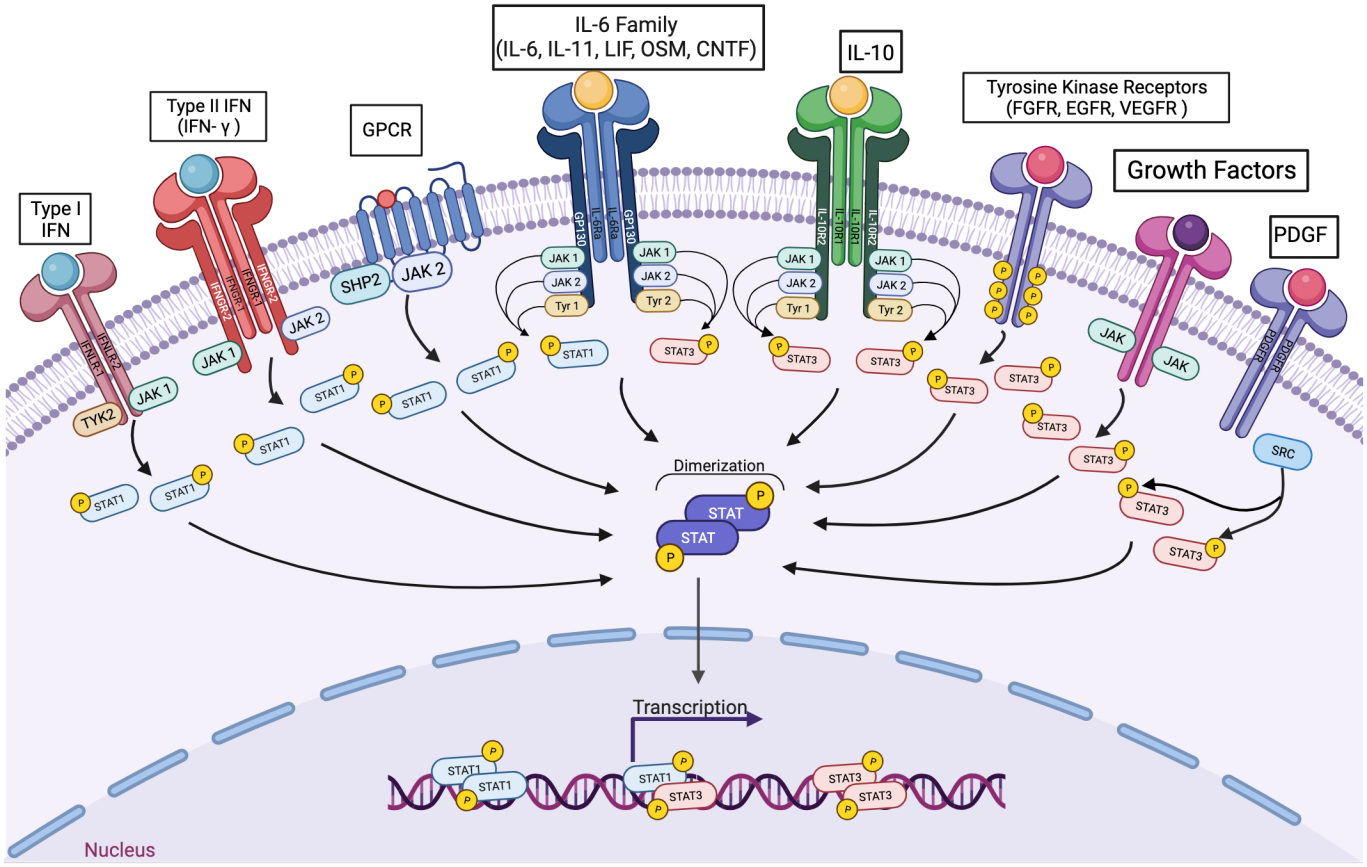
A wide variety of signaling pathways can lead to the activation of STAT1 or STAT3, individually and in combination. Cytokines interacting with receptors coupled to any jak family kinase can lead to the transcriptional activation of STAT1 homodimers, STAT3 homodimers, and/or STAT1-STAT3 heterodimers. In addition, polypeptide growth factor receptors and non-receptor tyrosine kinases can also mediate activating STAT3 phosphorylation.

The tyrosine phosphorylation of STATs can be mediated by a number of kinases. Since STATs are commonly activated downstream of cytokines whose receptors are coupled to jak family kinases, the term “JAK-STAT” pathway is commonly used to refer generally to these downstream events. However, given the diversity of genes regulated by the seven STAT family members, and the wide array of biological processes mediated by the STATs, this over-simplification can mask the broad array of biological events mediated by these transcription factors. In addition, STATs can be phosphorylated by non-JAK tyrosine kinases, further reflecting the fact that it is worthwhile to specify the effects of specific STATs (including STAT heterodimers) rather than grouping these all together (**Figure 1**).

**Figure 2:**
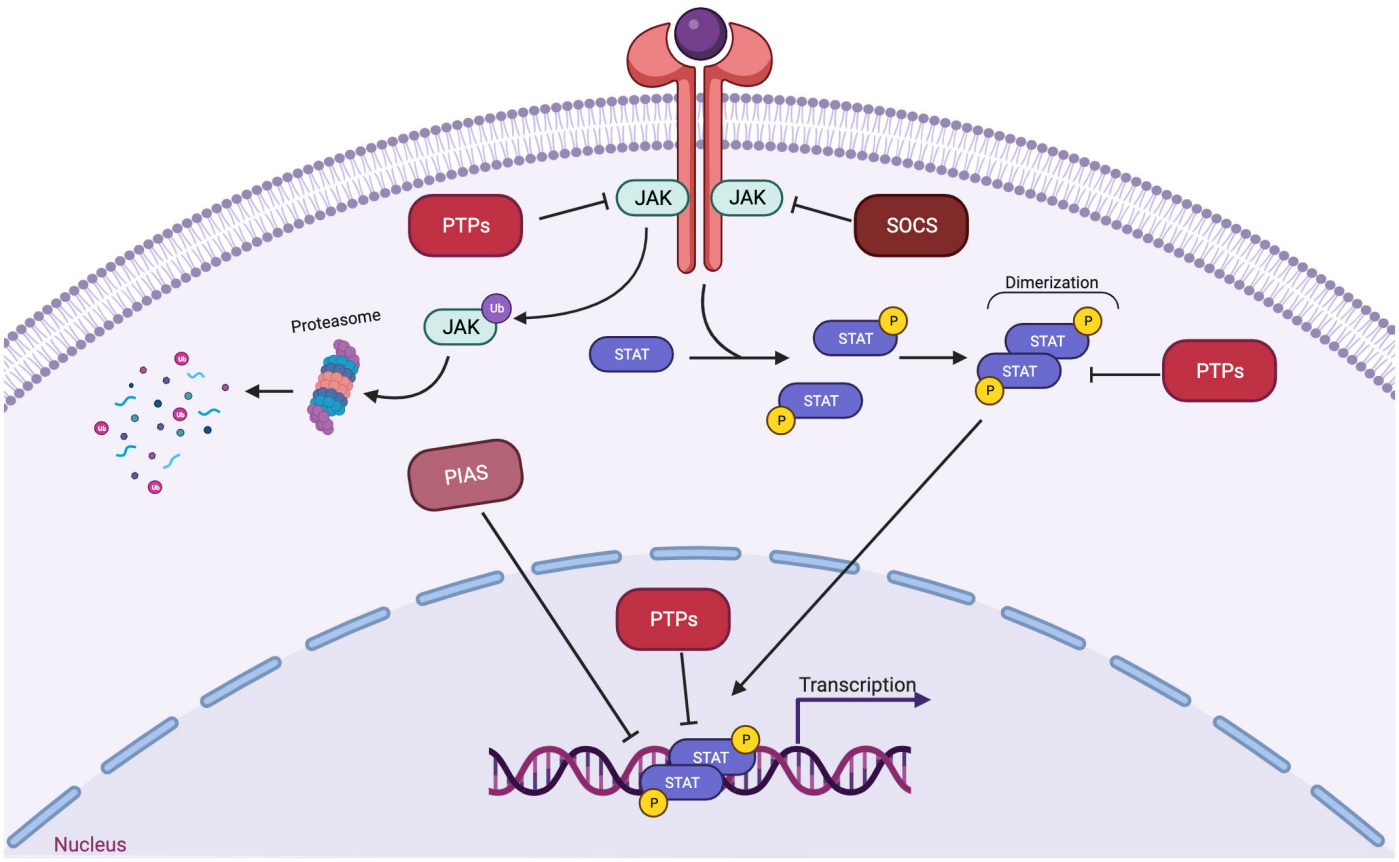
Three main families of proteins mediate negative regulation of STAT signaling. SOCS proteins, which includes CISH, are often transcriptionally induced by STATs, and suppress signaling at the level of the receptor-JAK complex. PIAS proteins interact with STAT dimers to inhibit STAT binding to DNA. PTPs (protein tyrosine phosphatases) dephosphorylate and inactivate JAK kinases and dephosphorylate and inactivate STAT dimers in both the cytoplasm and the nucleus.

In fact, more than 35 polypeptide ligands have been identified to activate STATs, including hormones, interferons (IFN), interleukins (ILs), and colony-stimulating factors (CSFs), many of which trigger opposing biological functions in the same cell (2) (**Table 1**). At the same time, signaling through the jak-STAT pathway is tightly controlled by several distinct mechanisms Just as there are many kinases and other mediators that can activate STAT transcriptional function, there are a wide range of phosphatases and other negative regulators that can dephosphorylate and inactivate STAT dimers, thus limiting STAT-dependent transcription (10) (**Figure 2**; **Table 2**). Key negative regulators of this pathway include suppressor of cytokine signaling (SOCS) proteins, phosphotyrosine phosphatases (PTPs), and protein inhibitors of activated STAT (PIAS). These molecules regulate JAK–STAT signaling at various steps through distinct mechanism (3). SOCS proteins appear to be the primary negative regulators of JAK and STAT signaling (14, 15). They negatively regulate these pathways by binding to phosphorylated tyrosine residues in receptor-kinase complexes to block the recruitment of STATs, inhibit JAK kinase activity, and target multiple components of the JAKs and their coupled receptors for ubiquitin-mediated proteasomal degradation (16–18). PTPs inhibit these pathways by interacting with JAKs, STATs, or receptors to dephosphorylate the STAT dimer and related JAKs and receptor components (8). PIAS proteins mainly interact with STAT dimers to inhibit STAT binding to DNA, thereby blocking STAT-dependent gene expression (19).

**Table 1:** Positive regulators of STAT signaling.

Positive regulators of STAT Signaling	References
**Serine kinases**	(3, 4)
MAPK1	
MAPK8	
MAPK14	
PRKCD	
CAMK2B	
**Cofactors**	(5–7)
BRCA1	
CREBBP	
EP300	
**Cooperating Transcription Factors**	(8, 9)
FOS	
JUN	
NFKB1	
NFKB2	
SMAD1	

**Table 2:** Negative regulators of STAT signaling.

Negative regulators of STAT Signaling	References
**PIAS**	(11)
PIAS1	
PIAS2	
PIAS3	
PIAS4	
**PTPs**	(12)
PTPRC	
PTPRE	
PTPN1	
PTPN2	
PTPN6	
PTPN11	
**SOCS**	(13)
SOCS1	
SOCS2	
SOCS3	
SOCS4	
SOCS5	
SOCS6	
SOCS7	

It should also be noted that STATs may play other roles in cellular function beyond their canonical role as transcription factors. STAT3, for example, can be phosphorylated on a serine residue located towards the carboxy terminus, and this phosphorylation may be important for roles of STAT3 in the mitochondria, which can affect cellular metabolism and energy flux (20).

## 3 STAT3 as a mediator of the physiologic acute phase response

STAT3 was first described as an important transcription factor in acute inflammation (21), and was found to control many normal or physiologic acute phase responses (22). In fact, this protein was originally named “acute phase response factor” or APRF. The acute phase response reflects an organism’s attempt to reestablish homeostasis after tissue injury, inflammation, or infection. Many cytokines released at sites of tissue injury, such as Interleukin 6 (IL-6), trigger STAT3 activation in viable cells in the microenvironment. STAT3 then activates transcription of genes regulating proliferation (such as cyclin D1), survival (such as BCL2 family members), and pluripotency (such as KLF4) as part of the physiologic response. Interestingly, many STAT3 target genes are themselves transcription factors, and thus STAT3 activation can trigger a cascade of biological programs (23) (**Table 3**). STAT3 activation also leads to the increased expression of immune suppressive proteins, such as PD-L1, and downregulation of co-stimulatory proteins. This affords protection from killing by infiltrating immune cells. As noted, there are multiple levels of negative regulators of STATs. Thus, while STAT-mediated gene expression can be detected within minutes of a stimulus, it is also shut off very quickly. This allows for tight regulation of the critical genes controlled by STAT3 and other family members.

**Table 3:** Downstream target genes of STAT3.

Gene	Function	Status	References
BATF	Differentiation	Upregulated	(24)
BCL6	Proliferation	Upregulated	(25)
c-MYC	Proliferation	Upregulated	(26)
CCL5	Immune Escape	Downregulated	(27)
CD80	Immune Escape	Downregulated	(28)
CD86	Immune Escape	Downregulated	(28)
CXCL10	Angiogenesis, Immune Escape	Downregulated	(29)
CCND1	Proliferation	Upregulated	(30)
FOSL2	Differentiation	Upregulated	(24)
GATA3	Differentiation	Upregulated	(24)
HIF1A	Angiogenesis	Upregulated	(31)
IFNB1	Angiogenesis, Immune Escape	Downregulated	(32)
IL10	Immune Escape	Upregulated	(33)
IL12	Angiogenesis, Immune Escape	Downregulated	(34)
IL6	Immune Escape	Upregulated	(27)
MMP2	Immune Escape	Upregulated	(35)
MMP9	Immune Escape	Upregulated	(36)
TP53	Proliferation	Downregulated	
RBPJ	Differentiation	Upregulated	(24)
RORA	Differentiation	Upregulated	(24)
STAT1	Differentiation	Downregulated	(24)
STAT3	Differentiation	Upregulated	(24)
BIRC5	Proliferation	Upregulated	
TGFB1	Immune Escape	Upregulated	(27)
TWIST1	Immune Escape	Upregulated	(37)
VEGF	Angiogenesis, Immune Escape	Upregulated	(38)
VIM	Immune Escape	Upregulated	(39)

Interestingly, STAT3 (and other STATs) can also participate in a positive feedback loop, since STAT promoters contain binding sites for their own dimers (40). This allows STATs to mediate amplified signals with repeated stimulation, and presumably evolved to accelerate and enhance the recovery from tissue injury.

## 4 STAT3 as an oncogenic transcription factor

Given that STAT3 target genes control processes that are known to underlie oncogenesis, such as proliferation, differentiation, survival, pluripotency, angiogenesis, invasion, and immune escape, it had been conjectured that inappropriate of constitutive activation of STAT3 might underlie cancer pathogenesis. In fact, it has been found that STAT3 (and other STAT family members, including STAT5) is activated commonly in a wide spectrum of human cancers. This can be detected by both direct detection of the activated, tyrosine-phosphorylated form of these proteins or by detection specific STAT-driven gene signatures (23). Furthermore, in the appropriate cellular contexts, STAT3 activation alone is sufficient to drive malignant cellular transformation (41). Reflecting the necessity of STAT3 in cancer pathogenesis, inhibition of STAT3, by genetic or pharmacologic means, inhibits the survival and proliferation of malignant cells in many experimental systems.

STAT3 can become activated constitutively through a variety of mechanisms that either drive increased phosphorylation of STAT3 or decreased inactivation. Among the factors driving increased STAT3 phosphorylation are mutations activating upstream tyrosine kinases or the increased presence of cytokines in a tumor microenvironment that can activate STAT3, produced through either autocrine or paracrine mechanisms (**Table 4**). Among such cytokines are IL-6, oncostatin M, and leukemia inhibitory factor (LIF). There is also evidence for a pathogenic role of a loss of negative regulators through genetic or epigenetic means, such as the silencing of the negative regulator of STAT3, suppressor of cytokine signaling 3 (SOCS3) (45).

**Table 4:** Identified mutations of STAT3.

Class	Genetic mutations	STAT3 protein expression	STAT3 phosphorylation	Disease	References
Autosomal dominant STAT3 loss-of-function	Point mutation (c.1282-89C>T)	Decrease	Decrease	STAT3 Hyper IgE Syndrome	(42)
Autosomal dominant STAT3 gain-of-function	Point mutation (c.1973A > T)	Increase	Increase	STAT3 gain-of-function syndrome	(43)
Acquired STAT3 gain-of-function	Frameshift insertion (exon1:c.264dupC), missense (exon13:c.C2210T)	Increase	Increase	Mature T-cell lymphomas	(44)

Although STAT3 is an oncogenic transcription factor, it is largely dispensable for normal cell functions (10). This suggests that STAT3 may be an attractive cancer therapeutic target, with a large therapeutic index (46).

## 5 STAT1 as a mediator of interferon signaling

In the late 1980s and early 1990s, as the role of STAT3 as a mediator of the acute phase response was being defined, parallel research was trying to elucidate the biological and transcriptional mediators of interferons. Interferons (IFNs) are a group of cytokines that activate a signal transduction cascade leading to the induction of hundreds of genes involved in antiviral defense, antiproliferative activities, and stimulation of adaptive immunity (47). There are three major types of IFNs: α, β, γ. Interferon α and β belong to the type I class of interferons, whereas interferon γ (IFN-γ) belongs to the type II class of interferons that is named “immune interferon.” IFN-γ is produced by cells of the immune system, including innate-like lymphocyte populations and adaptive immune cells (48). Type I interferons (IFN-α, IFN-β) stimulate the activity of TYK2 and JAK1, leading to the phosphorylation of STAT2. Then STAT2 forms a heterodimer with STAT1, which can enter the nucleus and activate transcription from target genes containing Interferon Stimulation Response Element (49). Signaling by the IFN-γ receptor induces receptor tyrosine phosphorylation by JAK1 and JAK2 proteins, producing a recruitment site for STAT1 (48). Activated STAT1 forms a homodimer, which then translocates to the nucleus and activates transcription from target genes containing Gamma-Activated Sequences (GAS) (50). Reflecting its critical role in mediating the effects of interferons on immune function, inherited STAT1 mutations have been shown to be associated with increased susceptibility to mycobacterial and fungal infections (**Table 5**).

**Table 5:** Identified mutations of STAT1.

Mutation	Genetic mutations	STAT1 protein expression	STAT1 phosphorylation	Disease	References
Autosomal recessive complete STAT1 deficiency	Deletion (1757–1758delAG), Substitution (T→C in exon 20), Frameshift (p.Val339ProfsTer18)	Null	Null	Lethal bacterial and viral diseases	(51, 52)
Autosomal recessive partial STAT1 deficiency	Missense (g.C2086T (P696S))	Decrease	Decrease	Curable bacterial and viral diseases	(53)
Autosomal dominant STAT1 deficiency	Substitution (L706S)	Normal	Decrease	Mycobacterial disease	(54)
Autosomal dominant STAT1 gain-of-function	Substitution (c.820C→T)	Increase	Increase	Chronic mucocutaneous candidiasis disease	(55)

## 6 STAT1 as a tumor suppressor

**Figure 3:**
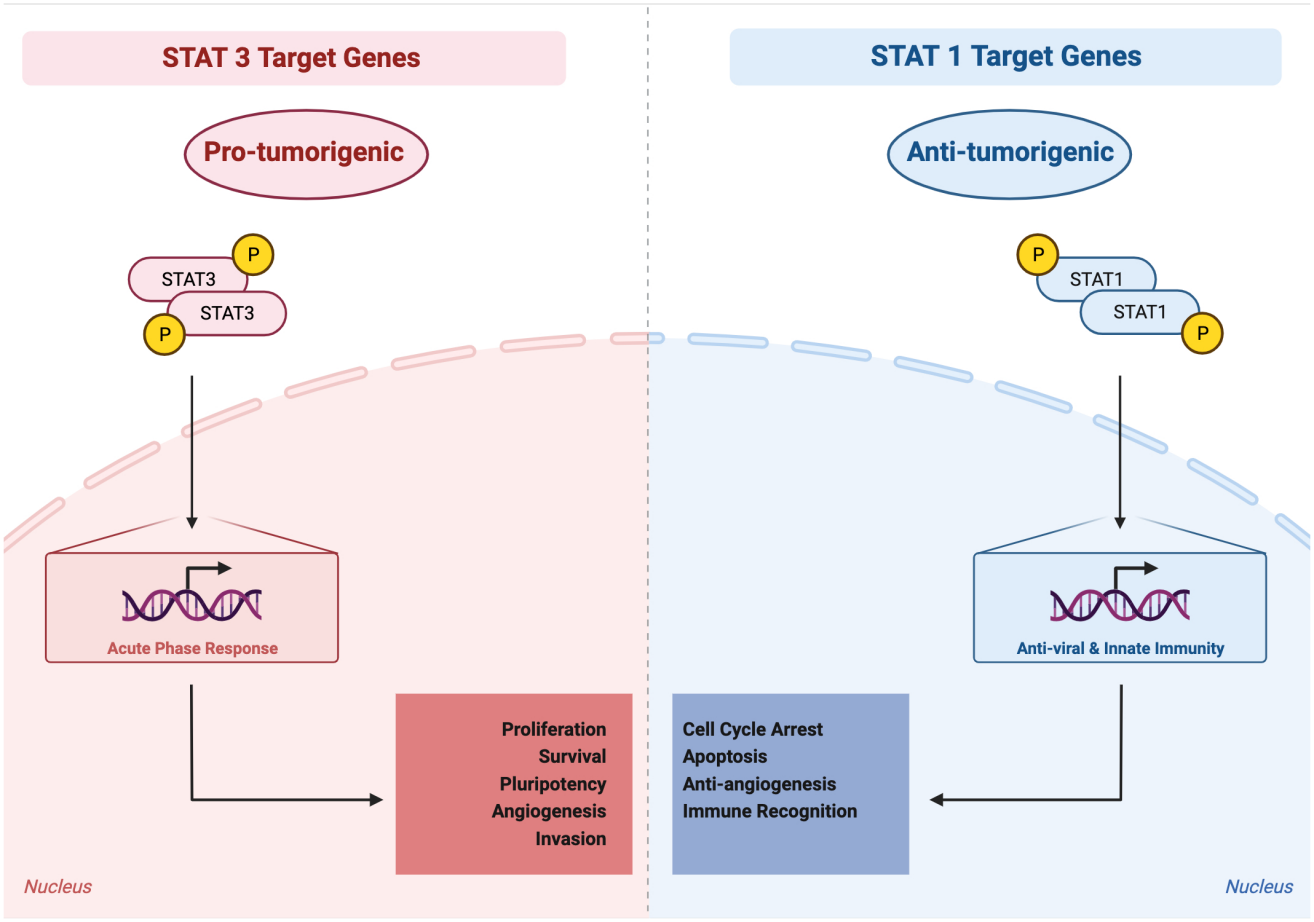
Activated STAT3 generally mediates pro-tumorigenic effects, whereas activated STAT1 generally mediates anti-tumorigenic effects. STAT3 target genes, reflecting their physiologic role in the acute phase response, regulate processes such as proliferation, survival, pluripotency, invasion, angiogenesis, and immune invasion. STAT1 target genes, reflecting their physiologic role in anti-viral and innate immunity, regulate processes such as cell cycle arrest, apoptosis, anti-angiogenesis, and immune recognition.

Reflecting its role in immunity, particularly anti-viral immunity, STAT1 generally acts as a tumor suppressor (**Figure 3**). This is true within tumor cells themselves, in immune cells, and in other cells in the tumor microenvironment, including endothelial cells. In tumor cells, STAT1 directs cytostatic and cytotoxic effects as well as immune stimulatory effects such as increased Major histocompatibility complex (MHC) expression. For example, in breast cancer cells, STAT1 signaling activated by IFN-γ and poly(I:C) can induce an increase in oxidative stress, potentiating the anti-tumor efficacy of the mitochondrial complex I inhibitor phenformin (56). In colon cancer cells, higher STAT1 expression is related to a significantly higher expression levels of MHC class I and PD-L1, which indicates a highly immunogenic microenvironment (57). Reduced tumor cell expression of STAT1 has been observed in many cancer types such as melanoma and chronic myeloid leukemia (58, 59).

In immune cells, STAT1 also acts to suppress tumor development and mediates anti-tumor effects. For example, in antigen presenting cells such as macrophages, dendritic cells, or B cells, STAT1 induced by IFN-γ is required for effective peptide recognition to increase the cell surface expression of MHC class II (60). In CD4 T-helper cells, signaling initiated by IFN-γ through STAT1 induces T-bet, which in turn stimulates further IFN-γ production to promote a feed-forward loop (61). In cytotoxic CD8 T cells, STAT1 mediates the upregulation of the pro-survival gene BCL2A1, which impairs the function of myeloid derived suppressor cells (62).

Furthermore, in endothelial cells, STAT1 induced by IFN-α mediates an anti-angiogenic effect in a Pml knock-out mouse model (63). Similarly, in isolated endothelial cells, STAT1 mediates anti-angiogenic effects (64).

Reflecting the context-dependence in transcription factor function, it should be noted that in certain conditions STAT1 may function as a tumor driver. For example, it has been found that STAT1 signaling stimulated by CD95/Fas is associated with an increase in cancer stemness in breast cancer cell lines (65).

Recognizing that STAT1 generally exerts anti-cancer effects, efforts have been made to identify small molecules that can amplify the transcriptional effect of STAT1. Using a high throughput screen of compounds that could enhance STAT1-dependent gene expression, a compound called 2-NP was identified as an enhancer of the inhibitory effect of IFN-γ on proliferation of tumor cells. As would be expected by its mechanism, 2-NP does not affect tumor cells lacking STAT1 (66).

## 7 Co-activation of STAT1 and STAT3 occurs commonly with cytokine stimulation

Although STAT3 and STAT1 mediate somewhat opposing effects in isolation, many cytokines activate both of these proteins simultaneously as part of their physiologic intracellular signaling. In particular, cytokines whose receptors share the common signaling protein gp130 can lead to co-activation of STAT1 and STAT3 (**Figure 1**). Gp130 (also known as glycoprotein 130, IL-6ST, IL-6R-β or CD130) is a transmembrane protein. It is composed of five fibronectin type-III domains and one immunoglobulin-like C2-type domain in its extracellular portion. Gp130 is ubiquitously expressed in mammalian cells. A wide variety of cytokines, many of which have been implicated in cancer pathogenesis, are known to signal through receptors that include gp130, such as IL-6, IL-11, LIF, and OSM. IL-6 and IL-11 initiate signaling via homodimerization of gp130, while LIF and OSM initiates signaling by heterodimerizing gp130 along with LIFR (67).

Gp130 can associate with any one of three of the four Jak family kinases, Jak1, Jak2, and Tyk2. Following cytokine engagement, gp130 associated with the cognate receptor undergoes a conformational change to bring the associated Jak kinases into juxtaposition, leading to activation of their tyrosine kinase activity. Gp130 then becomes phosphorylated on specific tyrosine residues, which can be recognized by the SH2 domain of both STAT1 and STAT3 (68). These STATs then become phosphorylated on their carboxy terminal activation tyrosine residues (Tyr 701 for STAT1 and Tyr 705 for STAT3) resulting in the formation of STAT1 homodimers, STAT3 homodimers, and STAT1:STAT3 heterodimers.

## 8 STAT1 and STAT3 have complex transcriptional dynamics

The cognate binding sites of STAT1 homodimers, STAT3 homodimers, and STAT1: STAT3 heterodimers are essentially identical in purified DNA, though there may be differences in genomic binding sites in the context of chromatin (69). Biologically, these dimers can oppose each other’s function and activation by a variety of mechanisms (70). For example, when co-activated by IL-6, STAT3 negatively regulates STAT1 by competing for common receptor docking sites and activating SOCS3 expression in mouse macrophages (71, 72). SOCS3 is a protein that can bind to STAT docking sites to regulate IL-6 signaling (73). Its deficiency results in prolonged activation of both STAT1 and STAT3 (74). In lung adenocarcinoma cells, OSM induces the inhibitory effect of the STAT1-dependent pathway and suppresses the activating effect of STAT3-dependent signaling, which, in combination, suppresses the expression of genes that regulate epithelial-mesenchymal transition and tumor metastasis (75).

In addition to gp130 family cytokines, other cytokines can also co-activate STAT1 and STAT3. In myeloid cells, IFN-α-activated STAT3 sequesters activated STAT1 to form heterodimers and prevents STAT1 from forming functional homodimers to transactivate downstream genes such as CXCL9 and CXCL10 (76). In some cellular systems, IFN-γ weakly activates STAT3, which suppresses formation of DNA-binding STAT1 homodimers and opposes the biological functions of STAT1 (76).

The final outcome of cytokine co-transactivation of STAT1 and STAT3 is distinct in different contexts, ranging from survival to apoptotic cell death or from inflammatory to anti-inflammatory responses (77). This partly reflects how specific cell types can integrate and interpret the complex and often contrasting signals they receive (78).

## 9 Targeting cytokines or Jaks has limited anti-cancer efficacy

**Figure 4:**
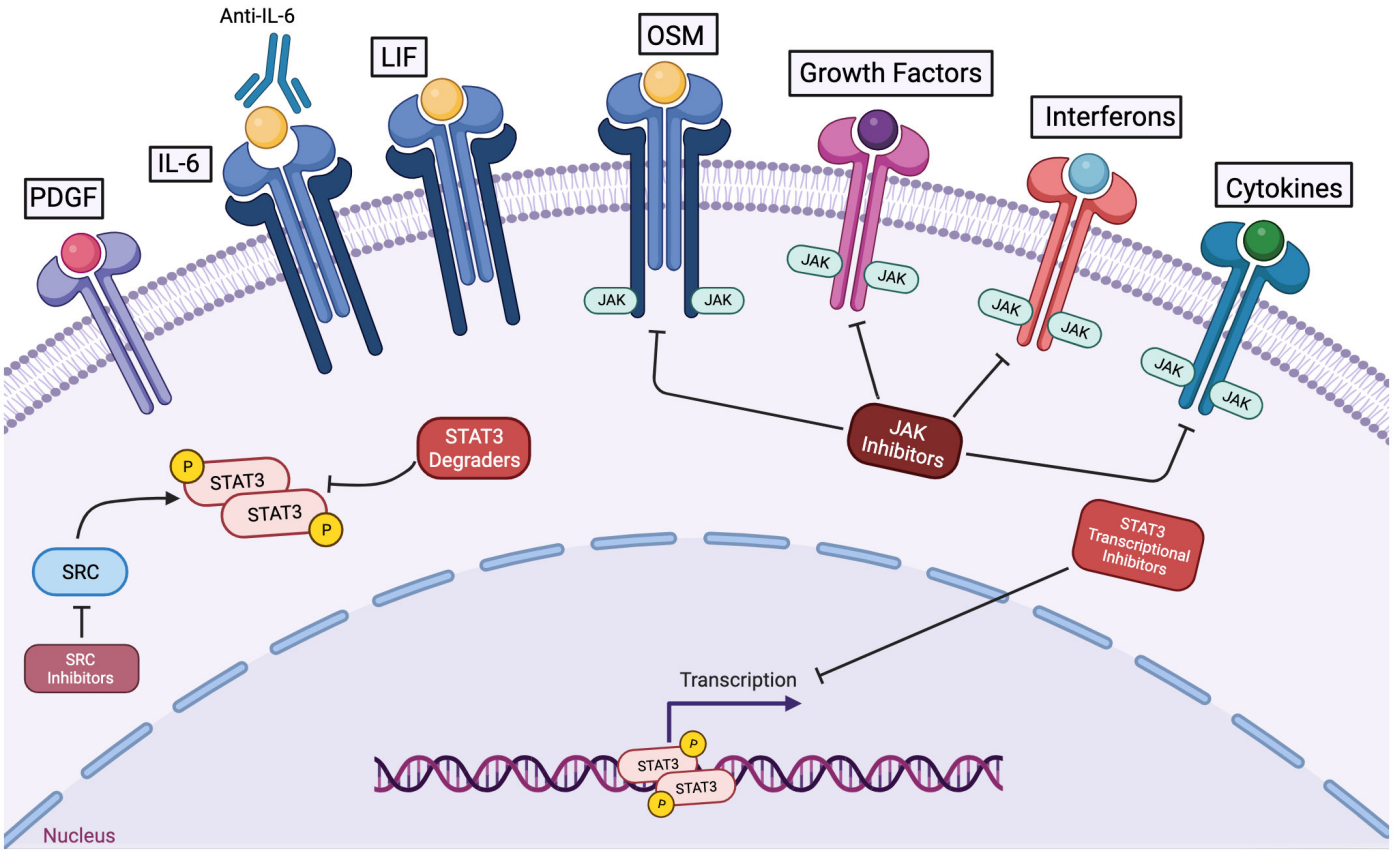
Among strategies to inhibit STAT3 function in cancer cells, STAT3 degraders and transcriptional inhibitors hold great potential to mediate anti-cancer effects without impairing the anti-tumor immune response or other cytokine-regulated processes. Antibodies to cytokines like IL-6 or to its receptor can block activation of STAT3 via this specific mechanism. However, in the tumor microenvironment, multiple pathways may contribute to STAT3 activation. Kinase inhibitors targeting jak family members can decrease cytokine-driven STAT3 activation, but they will not affect STAT3 activation mediated by other kinases. Furthermore, JAK inhibitors will block anti-tumor immune effects and the physiologic actions of other cytokines and growth factors. STAT3 degraders and STAT3 transcriptional inhibitors hold the promise of blocking the oncogenic effects of STAT3 without causing off-target or undesirable effects.

STAT3 is frequently activated inappropriately in cancer cells, and this is often driven, at least in part, by the presence of cytokines in the tumor microenvironment that can activate this protein, Consequently, there has been an interest in developing cancer therapeutics that block cytokines (either targeting the cytokines themselves or their cognate receptors) or Jak family kinases that are associated with cytokine receptors (**Figure 4**) (8).

The cytokine most often implicated in STAT3-driven cancer pathogenesis is IL-6. IL-6 has been found to be elevated in both the tumor microenvironment and systemically in many cancer models and in patients with a number of different forms of cancer. Furthermore, elevated IL-6 is associated with a worse prognosis in several cancers. It should be noted that IL-6 can be generated by a number of cells, and elevated systemic levels may reflect increased physiologic stress on an organism. This may be distinct from the role of IL-6 as a direct driver of STAT3 activation in the tumor microenvironment. Thus, elevated IL-6 may be a negative prognostic marker because it is reflecting the presence of a more advanced or aggressive tumor (79).

Interestingly, both IL-6 and another cytokine that signals through a gp130 receptor, OSM, have been associated with cancer cachexia, due to catabolic effects these cytokines have on muscle protein. These effects may reflect the role of these cytokines in generating the building blocks for tissue repair as part of the physiologic acute phase response following tissue injury, infection, and inflammation. A third cytokine that signals through gp130, LIF, also has been shown to play a role in the pathogenesis of cancers such as pancreatic cancer.

Perhaps reflecting the diversity of cytokines that can activate STAT3, therapies targeting individual cytokines have provided relatively little benefit in clinical trials. Antibodies both to the cytokine IL-6 and to the IL-6 receptor are FDA-approved for non-neoplastic indications, and have been tested in a number of clinical trials, generally in combination with other agents (80, 81). One potential reason for their limited efficacy is that multiple cytokines that can activate STAT3 (including OSM and LIF) may be present both in the tumor microenvironment and systemically, and blocking only one may have limited effects.

If targeting individual cytokines has limited therapeutic benefit in cancer, it is reasonable to consider whether targeting the next common downstream signaling protein, Jak family kinases, might provide a greater benefit. Small molecule JAK inhibitors that block the tyrosine kinase activity of jak family members may block the effect of multiple cytokines that play key roles in promoting cell proliferation, survival, and invasion (82). Several JAK inhibitors have been approved for clinical use, such as tofacitinib for the treatment of rheumatoid arthritis, ruxolitinib for atopic dermatitis, and pacritinib for myelofibrosis. Several of these drugs are in clinical trials for cancer treatment. For example, ruxolitinib (in conjunction with the PI3-kinase inhibitor parsaclisib) is being evaluated in patients with myelofibrosis (the LIMBER-313 trial). Ruxolitinib, in conjunction with the PI3 kinase inhibitor duvelisib, is being evaluated for the treatment of relapsed or refractory T- or NK-cell lymphoma (83). However, thus far there has been very little evidence for therapeutic benefit, and concerns have been raised that JAK inhibitors may be detrimental to cancer therapy. For example, a JAK inhibitor has been found to enhance metastatic burden in preclinical models of breast cancer by decreasing NK-cell-mediated anti-tumor immunity (84). A clinical trial evaluating ruxolitinib in advanced breast cancer also failed to show benefit (NCT01562873).

Although STAT3 activation in tumor cells is often (though not always) catalyzed by Jak family members, and Jak inhibitors may be effective at suppressing the activating tyrosine phosphorylation of STAT3, there are several reasons why Jak inhibitors may not provide much therapeutic benefit and may even have detrimental effects when used for cancer therapy. First, as noted, many of the cytokines that may be activating STAT3 in the tumor microenvironment are also activating STAT1. A JAK inhibitor will shut off both of these pathways. The net effect may be to remove the anti-proliferative and pro-apoptotic effects of STAT1. Furthermore, as noted earlier, STAT1 activation can be a key component of enhancing the immunogenicity of cancer cells by upregulating cell surface expression of histocompatibility proteins and other co-stimulatory molecules. STAT1 can enhance the anti-tumor effect of immune cells and it mediates anti-angiogenic effects in endothelial cells. Thus, it would be very important to understand how STAT1 signaling is influenced by anti-tumor treatment with JAK inhibitors (78, 85).

Since almost all cytokines, including those that enhance hematopoiesis and the immune response utilize subsets of the four Jak family members, even relatively selective Jak inhibitors tend to suppress both blood cell production and immune functioning. This is also likely a significant contributing factor to the limited efficacy, and potentially counterproductive effects of JAK inhibitors as components of anti-cancer regimens.

## 10 Novel therapeutic considerations

As discussed above, STAT1 and STAT3 generally play opposing roles in tumorigenesis, with STAT1 inhibiting tumorigenesis and STAT3 promoting tumorigenesis. Thus, therapeutic approaches that specifically downregulate STAT3 transcriptional activity and/or upregulate STAT1 transcriptional activity may be promising in cancer treatment (86) (**Tables 6**, **7**). One such example is trichothecin, a novel STAT3 inhibitor that was found to inhibit STAT3 activation and dimerization, but not affect phosphorylation levels of STAT1 (88). Tissue transglutaminase, an enzyme that crosslinks proteins between an ϵ-amino group of a lysine residue and a γ-carboxamide group of glutamine residue, is overexpressed in many cancer cells. The absence of this enzyme has been found to increase cytokine-induced STAT1 and attenuate STAT3 phosphorylation. This may promote T cell activation as a novel immunomodulatory target, and may also have direct anti-cancer or immune-sensitizing effects (110).

**Table 6:** Therapeutic Strategies to targeting STAT3.

Type	Agent	Indication	References
Small molecules	BP-1-102	NIH3T3/v-Src fibroblasts	(87)
	Trichothecin	Colorectal Cancer	(88)
	SF-1-066	NIH3T3/v-Src fibroblasts	(87)
	Stattic	Breast cancer	(89)
	S3I-201	Breast cancer	(90)
	STA-21	Breast cancer	(91)
Peptides	PS-acet.-STAT3 peptide	melanoma	(92)
Oligonucleotides	STAT3 double-stranded minicircles	Breast cancer	(93)
	TL13-112	Anaplastic Large Cell Lymphoma	(94)
	S3I-201	Gastric cancer	(95)
	SD-91	Megakaryoblastic leukemia	(96)
	TSM-1	head and neck squamous cell carcinoma and colorectal cancer	(97)
	C-STAT3DPROTAC	lymphoma	(98)
Combination therapy	Small-molecule STAT3 inhibitor (Napabucasin) and chemotherapy (FOLFIRI)	Metastatic Colorectal Cancer	(99)
	Small-molecule STAT3 inhibitor (Napabucasin) and chemotherapy (paclitaxel)	Advanced Gastric or Gastroesophageal Junction Adenocarcinoma	(100)
	STAT3 inhibitory peptide (APTSTAT3-9R) and immunotherapy (anti-PD-1 antibody)	vemurafenib-resistant melanoma	(101)

**Table 7:** Therapeutic strategies to targeting STAT1.

Type	Agent	Indication	STAT1 expression	References
MicroRNA	microRNA-370	Asthma	Increase	(102)
	microRNA-139-3p	Myocardial infarction	Increase	(103)
	lncRNA Sros1	Listeria monocytogenes	Increase	(104)
	miR-155	breast cancer	Increase	(105)
	miR130b	Lymphoma	Increase	(106)
	miR-146a-5p	Diabetic nephropathy	Increase	(107)
	miR-21-5p and miR-200a	Colorectal cancer	Decrease	(108)
Chemotherapy	Fludarabine	Esophageal squamous cell carcinoma	Decrease	(109)
	2NP	breast cancer and fibrosarcoma	Increase	(66)

STAT1 and STAT3 are also being explored for their tumor immunity-specific functions due to the increasing importance of immunotherapy, which refers to treatments that use the body’s own immune system to combat diseases. One form of cancer immunotherapy is checkpoint inhibitor therapy, which targets key regulators of the immune system. Programmed cell death-ligand 1 (CD274, PD-L1), which can be expressed on the surface of cancer cells binds to programmed cell death protein 1 (PDCD1, PD-1) on an immune cell surface, which inhibits immune cell activity (111, 112). While this PD-1/PD-L1 pathway has been targeted with some success in cancer therapy, many cancers fail to respond to PD-1 pathway blocking drugs or eventually develop resistance (113). It has been found that both INF-β-STAT3 and IFN-γ-STAT1 signaling pathways regulate PD-L1 expression (114). STAT1 or STAT3 knockdown by siRNA reduces cytokine-induced expression, but not constitutive PD-L1 expression in human monocytes and tumor cells (115). Selective inhibition of STAT1 and STAT3 by the JAK inhibitor ruxolitinib may improve the efficacy of anti-PD-1 immunotherapy (116). Other strategies are also being explored to modulate STAT1 and STAT3 transcriptional activity to enhance the efficacy of immune-based therapy. These approaches hold the promise of decreasing local tumor-induced immune suppression, and also making the cancer cells themselves more sensitive to immune-based killing.

A number of novel pharmacologic approaches have been taken to try to shift the STAT3-STAT1 equilibrium in a favorable direction for cancer therapy (**Figure 3**). One very direct approach to attenuate STAT3 signaling is to use targeted degraders of STAT3 to remove this protein from cells. In many systems, depletion of STAT3 by pharmacologic or genetic approaches enhances STAT1 activation. This may be from a direct stoichiometric effect, as STAT1 and STAT3 can bind to identical phosphorylated tyrosine residues in signaling proteins such as gp130, and the decreased competition for these sites from STAT3 allows a greater number of STAT1 proteins to become phosphorylated.

While most drug development efforts in this area have focused on inhibiting STAT3 transcriptional activity, as noted earlier, transcriptional screens have also identified molecules that can enhance STAT1-dependent transcription (66). It may be that a combination of these approaches will have the greatest therapeutic impact.

In summary, biological systems have complex homeostatic mechanisms that promote physiologic equilibrium. The common co-activation of STAT3 and STAT1, and their complex interactions likely evolved to promote the restoration of equilibrium in the setting of tissue injury and inflammation. While constitutive or inappropriate STAT3 activation is clearly a common pathogenic event in cancer development, and targeting this transcription factor can mediate therapeutic benefit, an understanding of these complex dynamics will likely allow a more refined and successful approach to cancer therapy.

## Author contributions

WW: Visualization, Writing – original draft, Writing – review & editing. ML, CK, MM, ZP, and CK: Visualization, Writing – review & editing. DF: Conceptualization, Funding acquisition, Supervision, Visualization, Writing – original draft, Writing – review & editing.
